# 1.B. Workshop: Adaptations in a time of crisis – public health practice in Sweden during the COVID-19 pandemic

**DOI:** 10.1093/eurpub/ckac129.004

**Published:** 2022-10-25

**Authors:** Organised by: The Public Health Agency of Sweden, Chair persons: Anna Månsdotter (Sweden), Karin Guldbrandsson (Sweden)

## Abstract

**Background:**

The Public Health Agency of Sweden has assessed the consequences of the COVID-19-pandemic in relation to the Swedish public health goals, aiming at good and equitable health. This assessment indicated 1) that preconditions for good and equitable health have changed during the pandemic, 2) that groups with higher risk of ill-health before the pandemic were more severely affected, 3) that the general health of the population remains good although some groups appear to have suffered from increased minor mental health problems, 4) that physical activity has decreased and sedentary behaviour has increased, and 5) that many activities of relevance for public health, such as prevention of alcohol, drugs, and tobacco (ADT) at the local and regional levels were paused or were replaced with other, often digital, alternatives.

**Objectives:**

The objective of this workshop is to present and discuss the results of four projects studying the consequences of the COVID-19-pandemic for public health practice in Sweden. Special attention is paid to ADT-prevention at the local and regional levels, focusing on how such work has developed digitally during the pandemic. Potential implications will be discussed with the audience. The added value of organizing this workshop: This workshop will contribute to an improved understanding of how public health practice, particularly ADT prevention, has navigated through a time of a crisis (i.e. a pandemic) in order to provide important lessons for the future. The presentations in this workshop will lay the foundation for discussing with the audience what might happen within the arena of health promotion and disease prevention as a result of a pandemic as well as ways to cope with the consequences of such a pandemic at the local and regional levels. The coherence between the presentations and the topic of the workshop: There is an obvious logic between the main topic of the workshop and the four presentations. In different ways, they all discuss consequences of the COVID-19-pandemic on public health practice in Sweden.

**The format of the workshop:**

The workshop will start with a brief introduction, aiming to give a short background and set the scene. Four presentations will then follow, with time allocated for questions after each presentation. The presentations will contain descriptions of each subproject, including the background, methods, results, and conclusions. Finally, the moderator will lead a discussion with the audience and the presenters.

**Key messages:**

• The pandemic forced public health practice to adapt its ways of working.

• When challenged, public health practice can operate in new ways, although with some regional differences.

